# Long-term association of the triglyceride-glucose index with cardiovascular involvement in systemic lupus erythematosus: a retrospective cohort study

**DOI:** 10.1136/lupus-2026-002141

**Published:** 2026-09-12

**Authors:** Zixian Deng, Jiayao Zheng, Yi Ye, Feng Chen, Huadong Liu, Xiaoyu Wang, Qiyun Liu, Chuangye Lyu, Cheng Liu, Lixiong Liu, Jie Yuan, Jianghua Li, Tangzhiming Li

**Affiliations:** 1 Department of Cardiology, Shenzhen People's Hospital (The First Affiliated Hospital, Southern University of Science and Technology; The Second Clinical Medical College, Jinan University), Shenzhen, 518020, People's Republic of China; 2 Department of Geriatrics, Shenzhen People's Hospital (The First Affiliated Hospital, Southern University of Science and Technology; The Second Clinical Medical College, Jinan University), Shenzhen, 518020, People's Republic of China; 3 Guangdong Provincial Clinical Research Center for Geriatrics, Shenzhen Clinical Research Center for Geriatrics, Department of Geriatrics, Shenzhen People's Hospital, Shenzhen, 518020, People's Republic of China; 4 Department of Cardiology, Shenzhen People's Hospital, The Second Clinical Medical College, Jinan University, Shenzhen, 518020, People's Republic of China; 5 Cardiology Department, Peking University First Hospital, Beijing, People's Republic of China; 6 Department of Cardiology, Long Gang Central Hospital of Shenzhen, Shenzhen, Guangdong, People's Republic of China; 7 Department of Rheumatology and Immunology, Shenzhen People’s Hospital (The First Affiliated Hospital, Southern University of Science and Technology; The Second Clinical Medical College, Jinan University), Shenzhen, 518020, People's Republic of China

**Keywords:** Systemic Lupus Erythematosus, Triglyceride–glucose Index, Cardiovascular Involvement, Inflammatory/lupus-related Cardiovascular Manifestations, Major Adverse Cardiovascular Events

## Abstract

**Background:**

SLE is a chronic autoimmune disease associated with heightened cardiovascular risk. However, the relationship between the triglyceride-glucose (TyG) index and cardiovascular involvement remains unclear. This study aimed to evaluate the association between the TyG index and cardiovascular involvement in SLE.

**Methods:**

We conducted a retrospective cohort study of 991 patients with SLE admitted between 2000 and 2021, spanning 21 years with extended follow-up. Kaplan-Meier curves were used to compare event-free survival across TyG quartiles, and differences between curves were assessed using the log-rank test. Time-to-event analyses were performed using Cox proportional hazards regression models. The proportional hazards assumption was assessed using scaled Schoenfeld residuals. Restricted cubic spline analyses based on Cox models were performed to evaluate the dose–response association between TyG and cardiovascular outcomes.

**Results:**

Cardiovascular involvement occurred in 251 patients (25.3%), consisting of 210 inflammatory or lupus-related manifestations and 41 major adverse cardiovascular events (MACE). Elevated TyG correlated with progressively lower event-free survival. An approximately linear association was observed between TyG and both overall cardiovascular involvement and inflammatory/lupus-related cardiovascular manifestations. Each one-unit increase in TyG remained associated with higher risk after multivariable adjustment (adjusted HR 1.36 for both outcomes, both p<0.05). These associations were materially unchanged after accounting for non-proportional covariate effects (overall cardiovascular involvement: HR 1.37, 95% CI 1.10 to 1.71; inflammatory/lupus-related manifestations: HR 1.35, 95% CI 1.06 to 1.74). For the original MACE composite, higher TyG was associated with increased risk in the adjusted analysis; however, this finding was less robust in a post hoc sensitivity analysis excluding heart failure and should be interpreted cautiously because of the limited number of events.

**Conclusion:**

These findings support the potential value of the TyG index as an accessible marker of cardiometabolic risk in SLE.

**WHAT IS ALREADY KNOWN ON THIS TOPIC**

- Patients with SLE experience substantially increased cardiovascular morbidity and mortality that cannot be fully explained by traditional cardiovascular risk factors alone. The triglyceride-glucose (TyG) index is a simple and inexpensive surrogate marker of insulin resistance and has been associated with cardiovascular events in the general population and several chronic inflammatory diseases. However, evidence regarding the long-term association between the TyG index and cardiovascular involvement in SLE remains limited.

**WHAT THIS STUDY ADDS**

- In this retrospective cohort study of 991 patients with SLE, higher TyG levels were associated with increased long-term cardiovascular involvement. The association remained evident after adjustment for traditional cardiovascular risk factors and available SLE-related clinical and laboratory characteristics and was also observed for inflammatory/lupus-related cardiovascular manifestations. An approximately linear dose–response association was identified for overall cardiovascular involvement and inflammatory/lupus-related cardiovascular manifestations, whereas findings for major adverse cardiovascular events were less robust.

**HOW THIS STUDY MIGHT AFFECT RESEARCH, PRACTICE OR POLICY**

- These findings support the further evaluation of the TyG index as an accessible marker of cardiometabolic risk in SLE. Prospective studies incorporating standardised measures of SLE activity and damage are needed to determine whether TyG provides clinically useful information for cardiovascular risk assessment.

## Introduction

 SLE is a chronic autoimmune disease characterised by immune dysregulation, persistent systemic inflammation and multisystem organ involvement. Despite substantial advances in diagnosis and treatment, SLE remains associated with considerable morbidity and premature mortality, particularly among young and middle-aged adults.^1 2^ As survival has improved over recent decades, long-term organ damage and comorbidity burden have become increasingly important determinants of patient outcomes.

Cardiovascular disease (CVD) is now recognised as one of the leading causes of death in patients with SLE. Compared with the general population, individuals with SLE experience substantially increased risks of myocardial infarction, stroke, heart failure and cardiovascular mortality.^3 4^ Importantly, cardiovascular involvement in SLE is not restricted to atherosclerotic disease but encompasses a broad spectrum of manifestations, including inflammatory cardiac injury, valvular abnormalities, arrhythmias, pulmonary vascular disease and ischaemic cardiovascular events.^5 6^ These cardiovascular complications contribute substantially to disease burden and long-term prognosis. Traditional cardiovascular risk factors alone do not fully explain the excess cardiovascular risk observed in SLE. In a landmark study, Esdaile *et al* demonstrated that conventional Framingham risk factors failed to account for the accelerated atherosclerosis observed in patients with SLE.^7^ Accumulating evidence suggests that chronic inflammation, immune activation, endothelial dysfunction and metabolic abnormalities may collectively contribute to cardiovascular damage in SLE.^8^ Therefore, identifying accessible biomarkers that capture both metabolic and disease-related cardiovascular risk remains an important clinical challenge.

The triglyceride-glucose (TyG) index, calculated from fasting triglyceride and fasting glucose concentrations, has emerged as a simple and reliable surrogate marker of insulin resistance.^9 10^ Compared with conventional measures such as the homeostasis model assessment of insulin resistance, the TyG index is inexpensive, reproducible and readily available in routine clinical practice. Increasing evidence has linked elevated TyG levels to adverse cardiovascular outcomes in the general population and in high-risk cardiometabolic cohorts. Previous studies have reported significant associations between TyG and coronary artery calcification, atherosclerotic plaque burden, heart failure and major adverse cardiovascular events (MACE).^9 11 12^ More recently, interest in the TyG index has expanded beyond traditional metabolic diseases. Studies in rheumatoid arthritis and psoriatic arthritis have suggested that TyG may also reflect cardiovascular vulnerability in chronic inflammatory disorders, supporting a potential interaction between insulin resistance and immune-mediated inflammation.^13–15^ These observations raise the possibility that TyG may have particular relevance in SLE, a disease characterised by both metabolic disturbances and persistent immune activation.

However, evidence regarding the association between TyG and cardiovascular involvement in SLE remains limited. Although insulin resistance has been linked to subclinical atherosclerosis and cardiovascular risk in SLE,^16^ the prognostic significance of the TyG index has not been adequately established. Existing studies have generally focused on cross-sectional associations, surrogate vascular markers or prediction models rather than longitudinal cardiovascular outcomes.^17^ Furthermore, the heterogeneous nature of cardiovascular manifestations in SLE has rarely been considered, and it remains unclear whether TyG is associated with both inflammatory cardiovascular manifestations and MACE.

Therefore, we conducted a retrospective cohort study of 991 patients with SLE to investigate the association between the TyG index and subsequent cardiovascular involvement. Using time-to-event analyses, restricted cubic spline modelling and outcome-specific analyses, we aimed to evaluate the longitudinal association between TyG and cardiovascular outcomes and to explore whether the observed association was consistent across different cardiovascular phenotypes in SLE.

## Methods

### Study population

This retrospective cohort study included patients with SLE admitted to Shenzhen People’s Hospital between January 2000 and December 2021. All data were anonymised before analysis to protect patient confidentiality. Because this was a retrospective observational study without additional patient intervention, the requirement for written informed consent was waived by the Ethics Committee. The waiver of written informed consent applied to the retrospective medical-record review. When supplementary telephone follow-up was required, patients or their family members were informed of the purpose of the contact and were free to decline further telephone follow-up.

**Figure 1:**
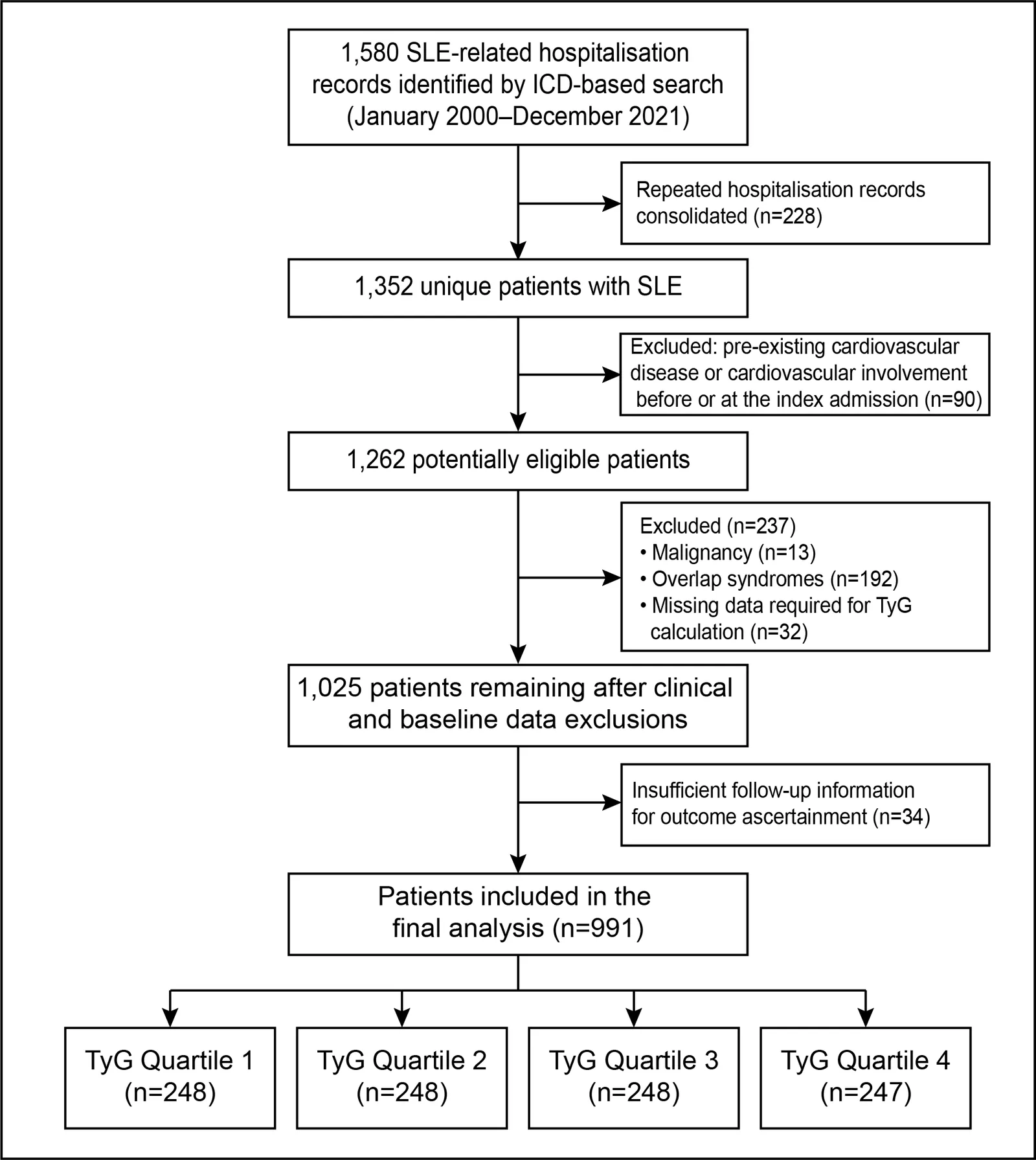
Study flowchart of patient selection and cohort assembly. ICD, International Classification of Diseases; TyG, triglyceride-glucose.

An International Classification of Diseases (ICD)-based search identified 1580 SLE-related hospitalisation records between January 2000 and December 2021. Records were linked using unique patient identifiers. After 228 repeated hospitalisation records were consolidated, 1352 unique patients with SLE remained. For patients with multiple hospitalisations, the first admission meeting the eligibility criteria and providing fasting triglyceride and glucose measurements was designated as the index admission. 90 patients with documented pre-existing CVD or cardiovascular involvement before or at the index admission were excluded, leaving 1262 potentially eligible patients. Among these patients, 13 were excluded because of malignancy, 192 because of concomitant autoimmune or overlap syndromes, and 32 because of missing data required for TyG calculation, leaving 1025 patients. A further 34 patients were excluded because insufficient follow-up information was available for reliable outcome ascertainment. The final analytical cohort therefore comprised 991 patients (figure 1).

Patients were eligible if they met the 1997 revised American College of Rheumatology (ACR) classification criteria for SLE and were aged ≥18 years. Patients were excluded if they had malignancy, concomitant autoimmune or overlap syndromes, missing triglyceride or fasting glucose data required for TyG calculation, pre-existing cardiovascular manifestations at baseline hospitalisation or invalid follow-up information. During the initial cohort screening, patients with documented pre-existing CVD or cardiovascular involvement before baseline were excluded based on available clinical records, medical history, electrocardiography, echocardiography, cardiovascular imaging when available and discharge diagnoses. The final analytical cohort therefore included patients without documented cardiovascular involvement before the baseline TyG assessment. Telephone follow-up was attempted when electronic medical records did not contain complete outcome information. Patients who declined participation during telephone follow-up were excluded from the final analysis. A total of 991 eligible patients were included in the final analytical cohort (figure 1).

### Data collection

Clinical data were retrospectively extracted from the hospital information system. Baseline was defined as the first hospitalisation at which fasting triglyceride and fasting glucose measurements were available for TyG calculation. Baseline variables included demographic characteristics, cardiometabolic risk factors, SLE-related clinical manifestations, medication use and laboratory findings.

Demographic and clinical variables included age, sex, smoking status, systolic blood pressure, diastolic blood pressure, heart rate, hypertension, hyperlipidaemia and diabetes mellitus. SLE-related manifestations included renal involvement, neuropsychiatric involvement, haematological involvement, arthritis and pleuritis. Renal involvement was defined according to the ACR renal criterion, including persistent proteinuria >0.5 g/day, >3+ proteinuria on urinalysis and/or biopsy-proven lupus nephritis. Haematological involvement included SLE-related anaemia, leucopenia, lymphopenia or thrombocytopenia based on laboratory and clinical assessment. Neuropsychiatric involvement, arthritis and pleuritis were identified based on medical records and physician documentation.

Laboratory variables included white blood cell count, platelet count, C reactive protein (CRP), erythrocyte sedimentation rate (ESR), serum creatinine, triglycerides, total cholesterol, high-density lipoprotein cholesterol, low-density lipoprotein cholesterol, complement C3, complement C4 and autoantibody profiles when available. Medication variables included glucocorticoid use. All laboratory values used for baseline analysis were obtained from the first eligible hospitalisation.

After identifying the previous inconsistency related to interstitial lung disease, we performed an additional data quality review for the main clinical variables included in the revised analyses. This review focused on demographic variables, cardiometabolic risk factors, SLE-related organ manifestations, inflammatory markers, complement levels, glucocorticoid use and cardiovascular outcome classifications. We checked variable definitions, coding consistency, missingness, plausible value ranges for continuous variables and frequency distributions for categorical variables. Key clinical variables and outcome classifications were further reviewed against the available medical records and discharge diagnoses when inconsistencies, implausible values or coding ambiguities were identified. Interstitial lung disease was not included as a covariate in the revised Cox models.

### TyG index calculation

The TyG index was calculated using the standard formula:

TyG index=ln (fasting triglycerides (mg/dL)×fasting glucose (mg/dL)/2).

For patients with multiple hospitalisations, baseline TyG was calculated using fasting triglyceride and fasting glucose values obtained during the first eligible hospitalisation. Because triglyceride and glucose values in the source dataset were recorded in mmol/L, unit conversions were performed before calculating TyG. Triglycerides were converted from mmol/L to mg/dL by multiplying by 88.57, and fasting glucose was converted from mmol/L to mg/dL by multiplying by 18. Patients with missing triglyceride or fasting glucose values were excluded from the analytical cohort. The TyG index was analysed both as a continuous variable and as quartiles based on its distribution in the analytical cohort, with quartile cutoffs of 8.06, 8.44 and 8.88.

### Outcomes

The outcome was overall cardiovascular involvement during follow-up. Incident cardiovascular involvement was identified through review of medical records, ECGs, echocardiography, coronary angiography, cardiac MRI and discharge diagnoses.

Overall cardiovascular involvement was defined as the occurrence of one or more of the following after baseline assessment: pericardial effusion, clinically relevant valvular disease, ventricular wall motion abnormality or hypertrophy, arrhythmia or conduction abnormality, ST-T abnormalities on resting 12-lead ECG, pulmonary hypertension suggested by echocardiography, heart failure, myocardial infarction, cerebral infarction or cardiovascular death. Physiological valvular regurgitation, congenital heart disease and cardiac abnormalities clearly attributable to non-SLE conditions were excluded where identifiable.

To address the heterogeneity of cardiovascular phenotypes in SLE, two secondary outcome categories were defined. Inflammatory or lupus-related cardiovascular manifestations were defined as cardiovascular involvement without MACE. MACE was defined as the occurrence of cardiovascular death, heart failure, cerebral infarction or myocardial infarction. Because the underlying aetiology of heart failure could not be uniformly adjudicated as ischaemic or atherosclerotic in this retrospective cohort, all incident heart failure events identified during follow-up were included in the original MACE composite. To assess the influence of this definition, we additionally performed a post hoc sensitivity analysis using a restricted MACE endpoint comprising cardiovascular death, cerebral infarction and myocardial infarction, with heart failure excluded. Patients were followed from baseline to the first occurrence of the outcome of interest, the last available clinical or telephone follow-up or 9 March 2022, whichever occurred first. Patients who remained free of the outcome of interest were right-censored at the date of their last available follow-up or at the administrative end of follow-up on 9 March 2022.

### Statistical analysis

Continuous variables were summarised as medians and IQRs, and categorical variables were summarised as frequencies and percentages. Baseline characteristics were compared across TyG quartiles using the Kruskal-Wallis rank-sum test for continuous variables and Pearson’s χ^2^ test or Fisher’s exact test for categorical variables, as appropriate.

Time zero for all time-to-event analyses was the date of the first eligible hospitalisation at which fasting triglyceride and fasting glucose measurements were simultaneously available for calculation of the baseline TyG index. Kaplan-Meier curves were used to compare event-free survival across TyG quartiles, and differences between curves were assessed using the log-rank test. Time-to-event analyses were performed using Cox proportional hazards regression models. HRs and 95% CIs were estimated for TyG both as a continuous variable and as quartiles, with the lowest quartile used as the reference group. Tests for trend across TyG quartiles were performed by modelling the quartile variable as an ordinal variable. To further evaluate model robustness, we summarised the number of patients, outcome events, regression parameters and events per parameter for each Cox model in online supplemental table S4.

For the primary outcome of overall cardiovascular involvement, three Cox models were constructed. Model 1 adjusted for age and sex. Model 2 additionally adjusted for smoking, hypertension, hyperlipidaemia and diabetes mellitus. Model 3 further adjusted for SLE-related manifestations and inflammatory or immunological markers, including renal involvement, neuropsychiatric involvement, haematologic involvement, arthritis, pleuritis, ESR, CRP, C3, C4 and glucocorticoid use. For inflammatory or lupus-related cardiovascular manifestations, a fully adjusted Cox model using the same covariates as Model 3 was applied. For MACE, because only 41 events occurred, a simplified adjusted model was used to limit model complexity and reduce the risk of overfitting. This model included a restricted set of clinically relevant covariates comprising age, sex, hypertension, hyperlipidaemia and diabetes mellitus. The MACE analyses were considered exploratory given the limited number of events.

Given the heterogeneous aetiology of heart failure in SLE, a post hoc sensitivity analysis was performed using a restricted MACE endpoint excluding heart failure and comprising cardiovascular death, cerebral infarction and myocardial infarction. Because only 24 restricted MACE events occurred, Cox models using continuous TyG were deliberately parsimonious. In addition to the crude model, analyses were adjusted for age and sex and, in the final sensitivity model, additionally for hypertension.

The proportional hazards assumption was assessed using variable-specific and global tests based on scaled Schoenfeld residuals, together with visual inspection of residual plots. The corresponding scaled Schoenfeld residual plots are presented in online supplemental figures 1 and 2. For models showing global non-proportionality, sensitivity analyses were performed to account for non-proportional effects among the adjustment covariates. Categorical covariates showing evidence of non-proportionality were incorporated as stratification variables, whereas continuous covariates were modelled using time-dependent coefficients through interactions with centred log follow-up time.

For overall cardiovascular involvement, age was modelled using a time-dependent coefficient, and the model was stratified by hyperlipidaemia and pleuritis. For inflammatory/lupus-related cardiovascular manifestations, age and C3 were modelled using time-dependent coefficients, and the model was stratified by hypertension, hyperlipidaemia and pleuritis. TyG itself satisfied the proportional hazards assumption in both models and was therefore retained as a time-invariant coefficient. Associations were re-estimated for continuous TyG, TyG quartiles and the ordinal trend across quartiles. Restricted cubic spline analyses based on Cox models were performed to evaluate the dose–response association between TyG and cardiovascular outcomes. Restricted cubic splines were fitted using four knots located at the fifth, 35th, 65th and 95th percentiles according to Harrell’s recommendations. The spline models for overall cardiovascular involvement and inflammatory or lupus-related manifestations were adjusted for age, sex, smoking, hypertension, hyperlipidaemia and diabetes mellitus. The MACE spline model was adjusted for age, sex, hypertension, hyperlipidaemia and diabetes mellitus. The p values for the overall association and for nonlinearity were reported.

All statistical analyses were conducted using R software. Data management and analyses were performed using the following R packages: readxl, dplyr, janitor, openxlsx, skimr, survival, survminer, broom, ggplot2, gtsummary, flextable, rms, forestplot, stringr, tidyr and purrr. Statistical tests were two-sided, and p<0.05 was considered statistically significant. Figures were exported at publication quality and further arranged when necessary for manuscript preparation.

## Results

### Study population and baseline characteristics

The ICD-based search identified 1580 SLE-related hospitalisation records. After consolidating 228 repeated hospitalisation records using unique patient identifiers, 1352 unique patients with SLE remained. 90 patients with pre-existing CVD or cardiovascular involvement before or at the index admission were excluded, leaving 1262 potentially eligible patients. A further 237 patients were excluded because of malignancy (n=13), concomitant autoimmune or overlap syndromes (n=192) or missing data required for TyG calculation (n=32). Of the remaining 1025 patients, 34 had insufficient follow-up information for reliable outcome ascertainment. Consequently, 991 patients were included in the final analysis (figure 1). The median age was 31 years (IQR 25–39 years), and 92.0% were female. The median follow-up time estimated using the reverse Kaplan-Meier method was 11.67 years (95% CI 11.23 to 12.38). During follow-up, 251 patients (25.3%) developed overall cardiovascular involvement, including 210 inflammatory/lupus-related cardiovascular manifestations and 41 MACE.

The median TyG index was 8.44 (IQR 8.06–8.88), and the quartile cutoffs were 8.06, 8.44 and 8.88. Baseline characteristics according to TyG quartiles are presented in table 1. Patients in the highest TyG quartile exhibited a less favourable cardiometabolic profile, with significantly higher prevalences of smoking, hypertension, hyperlipidaemia and diabetes mellitus (all p<0.05). Higher TyG levels were also associated with a greater burden of SLE-related organ involvement, including renal involvement, neuropsychiatric involvement, arthritis and pleuritis (all p<0.001). Patients with higher TyG levels exhibited higher ESR and less favourable lipid profiles, together with lower complement levels. CRP concentrations also varied significantly across TyG quartiles (all p<0.001). The proportion of patients receiving glucocorticoid therapy did not differ significantly among quartiles.

**Table 1:** Baseline characteristics of patients with SLE according to triglyceride-glucose index quartiles

Characteristic	Overall n=991	Q1 (≤8.06) n=248	Q2 (>8.06 to ≤8.44) n=248	Q3 (>8.44 to ≤8.88) n=248	Q4 (>8.88) n=247	P value
Demographics						
Age	31 (25–39)	30 (25–37)	32 (26–41)	31 (24–40)	30 (24–40)	0.173
Sex						0.216
Female	912 (92%)	236 (95%)	225 (91%)	226 (91%)	225 (91%)	
Male	79 (8.0%)	12 (4.8%)	23 (9.3%)	22 (8.9%)	22 (8.9%)	
Smoking	16 (1.6%)	1 (0.4%)	2 (0.8%)	3 (1.2%)	10 (4.0%)	0.011
Systolic BP	110 (101–123)	108 (101–119)	110 (101–122)	110 (99–124)	113 (101–128)	0.247
Diastolic BP	74 (66–83)	74 (68–80)	74 (67–82)	74 (64–84)	75 (66–87)	0.540
Heart rate	88 (80–100)	87 (80–98)	87 (78–97)	89 (80–100)	90 (82–102)	0.002
Cardiometabolic factors						
Hypertension	170 (17%)	26 (10%)	33 (13%)	49 (20%)	62 (25%)	<0.001
Hyperlipidaemia	77 (7.8%)	4 (1.6%)	11 (4.4%)	16 (6.5%)	46 (19%)	<0.001
Diabetes	29 (2.9%)	0 (0%)	6 (2.4%)	3 (1.2%)	20 (8.1%)	<0.001
SLE manifestations						
Kidney involvement	93 (9.4%)	12 (4.8%)	16 (6.5%)	26 (10%)	39 (16%)	<0.001
Neuropsychiatric involvement	295 (30%)	56 (23%)	65 (26%)	75 (30%)	99 (40%)	<0.001
Haematological involvement	94 (9.5%)	17 (6.9%)	20 (8.1%)	29 (12%)	28 (11%)	0.174
Arthritis	257 (26%)	37 (15%)	56 (23%)	76 (31%)	88 (36%)	<0.001
Pleuritis	299 (30%)	54 (22%)	57 (23%)	87 (35%)	101 (41%)	<0.001
Laboratory findings						
White blood cells	5.10 (3.58–7.10)	5.30 (3.76–7.60)	5.11 (3.59–7.05)	4.96 (3.46–6.65)	4.94 (3.46–7.15)	0.207
Platelets	195 (136–253)	210 (158–259)	186 (136–247)	194 (131–259)	185 (113–245)	0.027
C reactive protein	3 (1–11)	2 (1–7)	3 (1–12)	3 (1–12)	3 (1–11)	<0.001
Erythrocyte sedimentation rate	38 (16–68)	22 (10–50)	33 (14–57)	41 (20–79)	50 (28–80)	<0.001
Complement 3	0.66 (0.43–0.91)	0.74 (0.57–0.92)	0.71 (0.50–0.94)	0.63 (0.39–0.92)	0.49 (0.31–0.81)	<0.001
Complement 4	0.12 (0.06–0.19)	0.14 (0.08–0.19)	0.13 (0.06–0.19)	0.12 (0.06–0.18)	0.07 (0.05–0.16)	<0.001
Creatinine	60 (52–71)	58 (51–66)	60 (52–70)	59 (51–73)	63 (53–84)	<0.001
Total cholesterol	4.09 (3.35–4.92)	3.84 (3.27–4.47)	4.03 (3.27–4.78)	4.13 (3.35–4.94)	4.51 (3.69–5.62)	<0.001
High-density lipoprotein-cholesterol	1.01 (0.76–1.32)	1.23 (0.97–1.53)	1.06 (0.83–1.34)	0.93 (0.72–1.15)	0.86 (0.65–1.12)	<0.001
Low-density lipoprotein cholesterol	2.26 (1.76–2.89)	2.05 (1.67–2.50)	2.25 (1.77–2.84)	2.37 (1.86–2.94)	2.44 (1.89–3.29)	<0.001
Treatment						
Glucocorticoid	752 (76%)	183 (74%)	178 (72%)	197 (79%)	194 (79%)	0.136
Outcomes						
Overall cardiovascular involvement	251 (25%)	39 (15.7%)	57 (23%)	70 (28.2%)	85 (34.4%)	<0.001
Inflammatory or lupus-related cardiovascular manifestations	210 (21%)	35 (14.1%)	48 (19.4%)	61 (24.6%)	66 (26.7%)	0.003
Major adverse cardiovascular events	41 (4.1%)	4 (1.6%)	9 (3.6%)	9 (3.6%)	19 (7.7%)	0.007

Data are presented as n (%) or median (IQR).

BP, blood pressure.

Importantly, the incidence of cardiovascular outcomes increased progressively across TyG quartiles. Overall cardiovascular involvement occurred in 15.7%, 23.0%, 28.2% and 34.4% of patients in Q1–Q4, respectively (p<0.001). Similar trends were observed for inflammatory/lupus-related manifestations (14.1%, 19.4%, 24.6% and 26.7%; p=0.003) and MACE (1.6%, 3.6%, 3.6% and 7.7%; p=0.007).

### Kaplan-Meier analysis of cardiovascular outcomes

**Figure 2:**
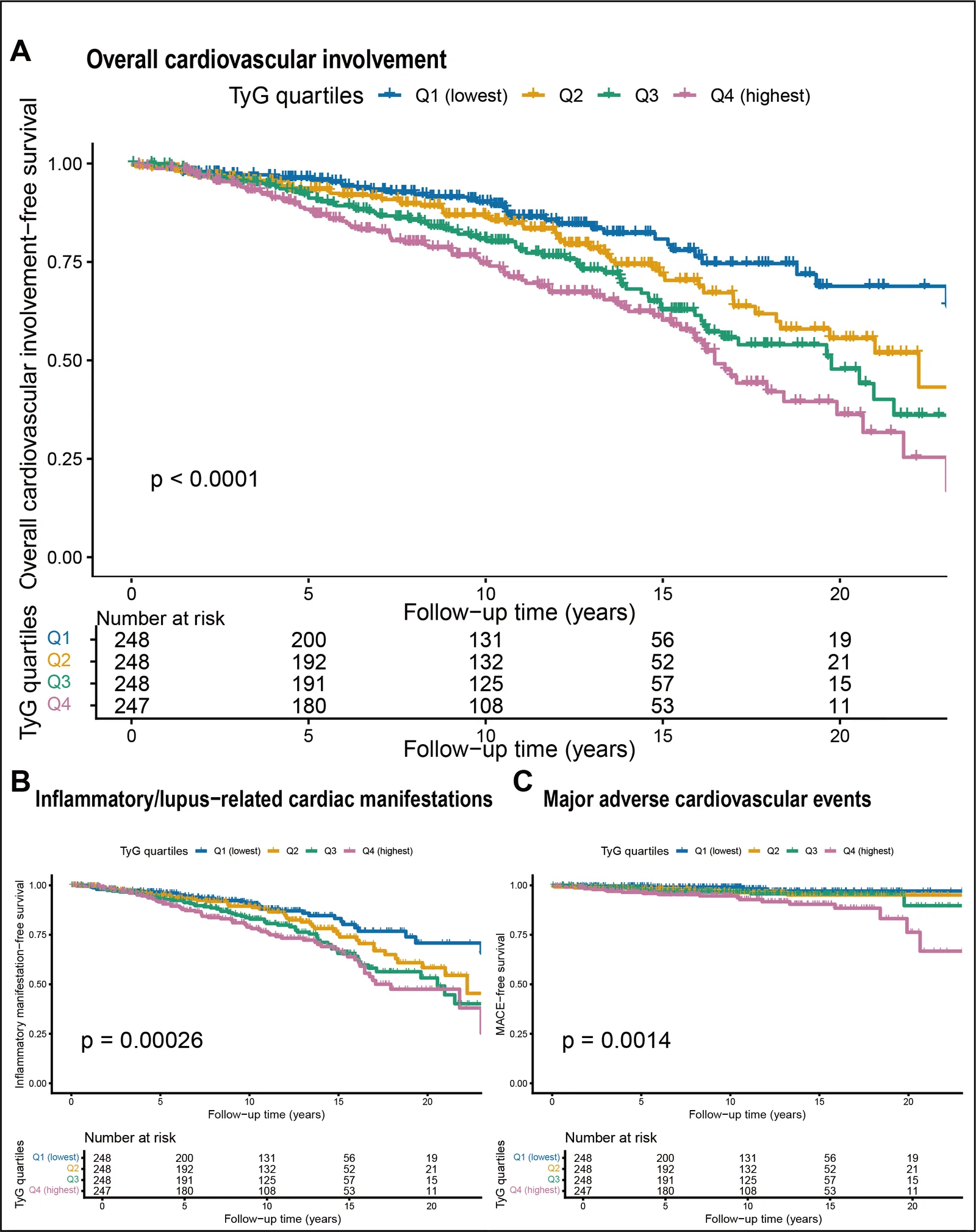
Kaplan-Meier curves for cardiovascular outcomes according to TyG index quartiles. Kaplan-Meier estimates of event-free survival according to baseline TyG index quartiles. Panel A shows overall cardiovascular involvement. Panel B shows inflammatory/lupus-related cardiovascular manifestations. Panel C shows major adverse cardiovascular events. Differences among TyG quartiles were assessed using the log-rank test. Tick marks indicate censored observations. Numbers at risk are shown below each panel. TyG, triglyceride-glucose.

Kaplan-Meier analyses demonstrated significant differences in event-free survival across TyG quartiles (figure 2). For overall cardiovascular involvement, cumulative event-free survival progressively declined with increasing TyG levels, with patients in Q4 showing the highest incidence of events throughout follow-up (log-rank p<0.0001; figure 2A). A similar pattern was observed for inflammatory/lupus-related cardiovascular manifestations, with significantly lower event-free survival among patients with higher TyG values (log-rank p=0.00026; figure 2B). For MACE, patients in the highest TyG quartile also experienced poorer event-free survival compared with lower quartiles (log-rank p=0.0014; figure 2C).

### Dose–response relationship between TyG and cardiovascular outcomes

**Figure 3:**
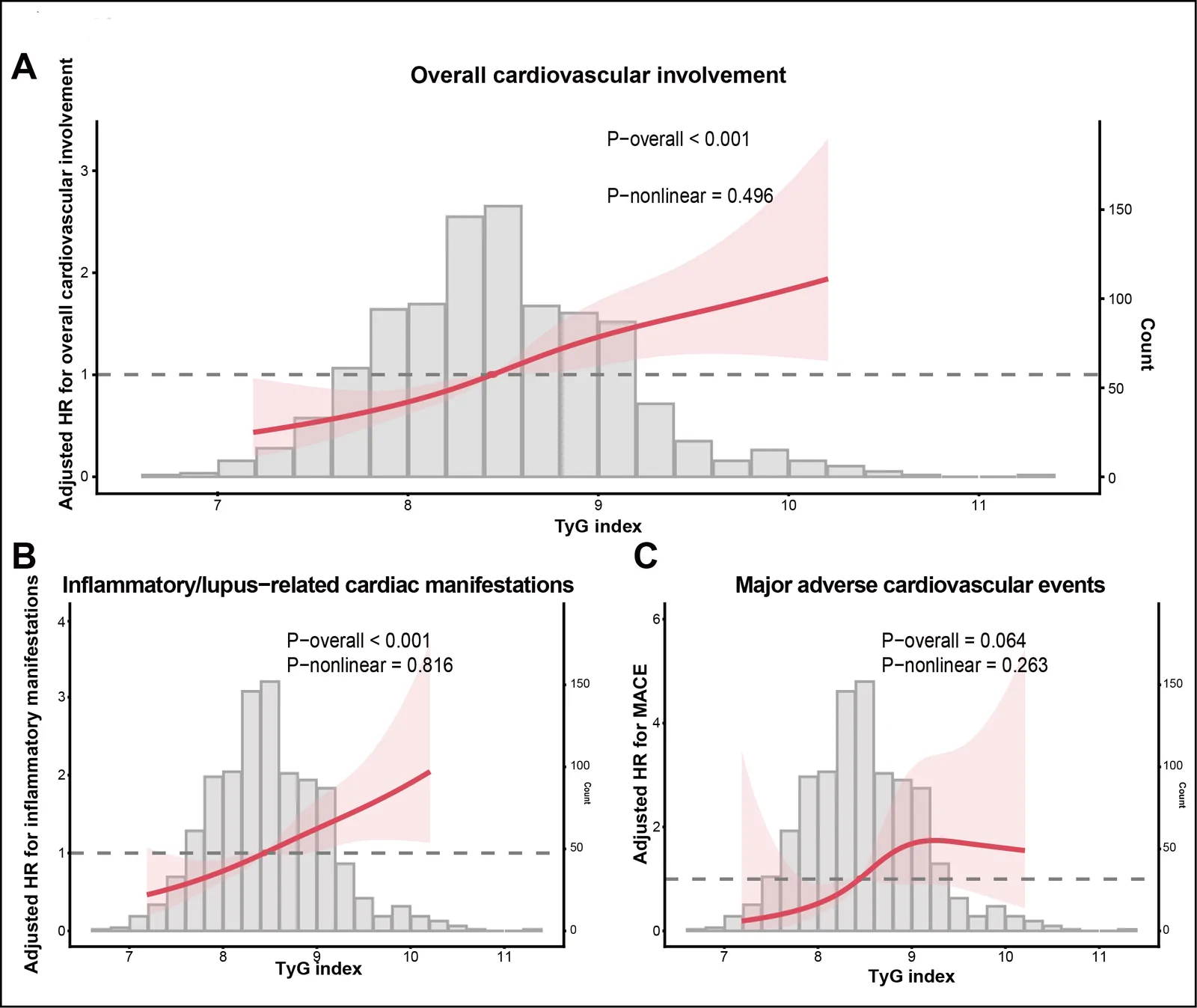
Restricted cubic spline analyses of the association between the TyG index and cardiovascular outcomes. Restricted cubic spline analyses illustrating the dose–response relationship between the TyG index and cardiovascular outcomes. Panel A shows overall cardiovascular involvement. Panel B shows inflammatory/lupus-related cardiovascular manifestations. Panel C shows major adverse cardiovascular events. Solid red lines represent adjusted HRs, and shaded areas represent 95% CIs. Histograms indicate the distribution of TyG values within the study population. The dashed horizontal line indicates an HR of 1.0. The p values for the overall association and for nonlinearity are displayed within each panel. TyG, triglyceride-glucose

Restricted cubic spline analyses were performed to investigate the shape of the association between TyG and cardiovascular outcomes (figure 3). For overall cardiovascular involvement, a significant positive dose–response relationship was observed (P-overall<0.001), while no evidence of nonlinearity was detected (P-non-linear=0.496), suggesting an approximately linear increase in risk across the TyG spectrum (figure 3A). Similarly, TyG demonstrated a significant linear association with inflammatory/lupus-related cardiovascular manifestations (P-overall<0.001; P-non-linear=0.816; figure 3B). For MACE, the overall association did not reach conventional statistical significance (P-overall=0.064), although a trend toward increased risk at higher TyG levels was observed (figure 3C).

### Cox regression analyses

**Figure 4:**
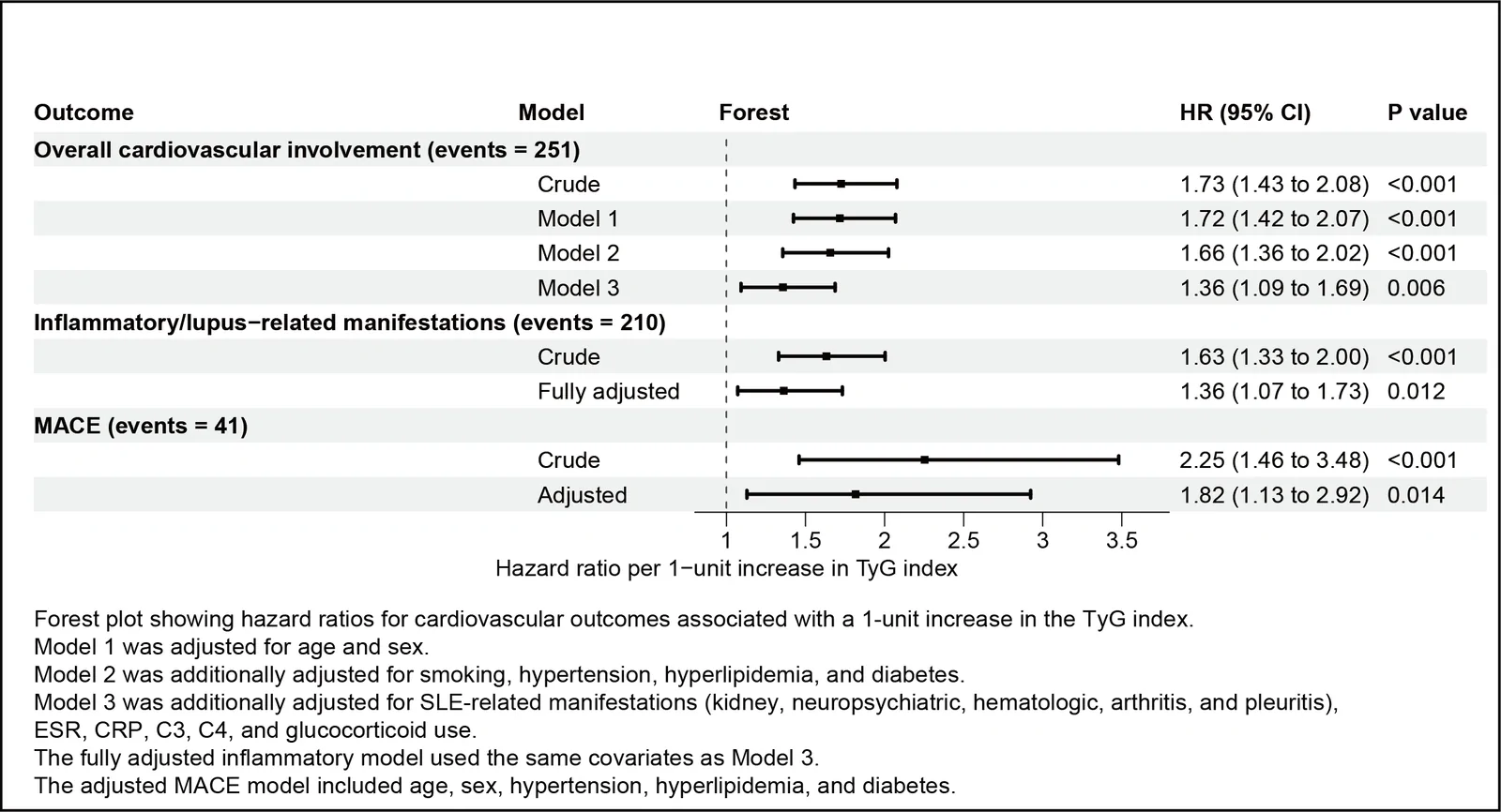
Forest plot showing the association of a one-unit increase in the TyG index with cardiovascular outcomes. Forest plot showing HRs and 95% CIs associated with each one-unit increase in the TyG index for overall cardiovascular involvement, inflammatory/lupus-related cardiovascular manifestations and MACE. Crude and adjusted Cox proportional hazards models are presented. The vertical dashed line indicates an HR of 1.0. Detailed covariate adjustment for each model is described in the Methods section and table 2. C3, complement 3; C4, complement 4; CRP, C reactive protein; ESR, erythrocyte sedimentation rate; MACE, major adverse cardiovascular events; TyG, triglyceride-glucose.

The association between the TyG index and cardiovascular outcomes was further evaluated using Cox proportional hazards models (figure 4 and table 2). For overall cardiovascular involvement, each one-unit increase in TyG was associated with a significantly increased risk in the crude model (HR 1.73, 95% CI 1.43 to 2.08; p<0.001). The association remained significant after adjustment for age and sex (Model 1: HR 1.72, 95% CI 1.42 to 2.07; p<0.001), additional cardiometabolic risk factors (Model 2: HR 1.66, 95% CI 1.36 to 2.02; p<0.001) and SLE-related manifestations, inflammatory markers, complement levels and glucocorticoid use (Model 3: HR 1.36, 95% CI 1.09 to 1.69; p=0.006). For inflammatory/lupus-related cardiovascular manifestations, the crude HR was 1.63 (95% CI 1.33 to 2.00; p<0.001), and the association remained significant after full adjustment (HR 1.36, 95% CI 1.07 to 1.73; p=0.012). For MACE, each one-unit increase in TyG was associated with a more than twofold higher risk in the crude model (HR 2.25, 95% CI 1.46 to 3.48; p<0.001). After adjustment for age, sex, hypertension, hyperlipidaemia and diabetes mellitus, the association remained statistically significant (HR 1.82, 95% CI 1.13 to 2.92; p=0.014).

**Table 2:** Cox regression analyses for overall cardiovascular involvement according to the TyG index

Outcome	Exposure	Model 1 hour (95% CI)	Model 1 P value	Model 2 hour (95% CI)	Model 2 P value	Model 3 hour (95% CI)	Model 3 P value
Overall cardiovascular involvement	Continuous TyG	1.72 (1.42 to 2.07)	<0.001	1.66 (1.36 to 2.02)	<0.001	1.36 (1.09 to 1.69)	0.006
Overall cardiovascular involvement	TyG quartiles						
Overall cardiovascular involvement	Q1	Ref.		Ref.		Ref.	
Overall cardiovascular involvement	Q2	1.52 (1.01 to 2.29)	0.045	1.56 (1.03 to 2.35)	0.035	1.29 (0.84 to 1.96)	0.246
Overall cardiovascular involvement	Q3	1.92 (1.29 to 2.85)	0.001	1.87 (1.25 to 2.79)	0.002	1.55 (1.03 to 2.33)	0.036
Overall cardiovascular involvement	Q4	2.66 (1.82 to 3.91)	<0.001	2.56 (1.72 to 3.81)	<0.001	1.68 (1.12 to 2.54)	0.013
Overall cardiovascular involvement	P for trend		<0.001		<0.001		0.009

Model 1 adjusted for age and sex.

Model 2 additionally adjusted for smoking, hypertension, hyperlipidaemia, and diabetes.

Model 3 additionally adjusted for kidney involvement, neuropsychiatric involvement, haematological involvement, arthritis, pleuritis, erythrocyte sedimentation rate, C reactive protein, complement 3, complement 4 and glucocorticoid use.

TyG, triglyceride-glucose; TyG, triglyceride-glucose.

In a post hoc sensitivity analysis excluding heart failure from the MACE composite, 24 restricted MACE events comprising cardiovascular death, cerebral infarction and myocardial infarction remained. The association of continuous TyG with the restricted endpoint remained positive in crude and age- and sex-adjusted models (HR 2.07, 95% CI 1.15 to 3.72, p=0.015; and HR 2.08, 95% CI 1.14 to 3.81, p=0.018, respectively), but was attenuated after additional adjustment for hypertension (HR 1.57, 95% CI 0.85 to 2.89, p=0.147). The proportional hazards assumption showed evidence of deviation for TyG in this restricted analysis (p=0.029), and these findings were therefore regarded as exploratory (online supplemental table S3).

Quartile-based analyses further demonstrated an overall gradient in cardiovascular risk across increasing TyG categories, although the patterns differed among outcomes. For overall cardiovascular involvement, the hazard increased from Q2 to Q4, with Q4 showing a significantly higher risk than Q1 in the fully adjusted model (HR 1.68, 95% CI 1.12 to 2.54; p=0.013; p for trend=0.009) (table 2). For inflammatory/lupus-related cardiovascular manifestations, the highest estimates were observed in Q3 and Q4, although the Q4 estimate was of borderline statistical significance (Q3: HR 1.56, p=0.045; Q4: HR 1.55, p=0.053) (online supplemental table S1A). For the original MACE composite, the highest TyG quartile was associated with increased risk in the simplified adjusted model (HR 4.67, 95% CI 1.51 to 14.43; p=0.007), whereas estimates for the intermediate quartiles were not statistically significant (online supplemental table S1B).

Scaled Schoenfeld residual tests indicated that TyG satisfied the proportional hazards assumption for both overall cardiovascular involvement (p=0.552) and inflammatory/lupus-related cardiovascular manifestations (p=0.821). However, the global tests indicated non-proportionality in the corresponding fully adjusted models (global p=0.010 and p=0.001, respectively). Evidence of non-proportionality was observed for age, hyperlipidaemia and pleuritis in the overall cardiovascular involvement model and for age, hypertension, hyperlipidaemia, pleuritis and C3 in the inflammatory/lupus-related manifestations model. (online supplemental table S2) and online supplemental figures 1 and 2.

After accounting for these non-proportional covariate effects, each one-unit increase in TyG remained associated with overall cardiovascular involvement (HR 1.366, 95% CI 1.095 to 1.705; p=0.006) and inflammatory/lupus-related cardiovascular manifestations (HR 1.354, 95% CI 1.055 to 1.737; p=0.017). These estimates differed from the corresponding conventional Cox estimates by less than 1%.

In the proportional hazards-adjusted quartile analysis, the association remained evident for overall cardiovascular involvement (Q4 vs Q1: HR 1.616, 95% CI 1.069 to 2.443; p=0.023; p for trend=0.014). For inflammatory/lupus-related manifestations, the quartile estimates remained directionally consistent, although the trend estimate was attenuated and no longer reached conventional statistical significance (HR per quartile increment 1.141, 95% CI 0.992 to 1.313; p for trend=0.065). Detailed comparisons between the conventional and proportional hazard-adjusted models for continuous TyG and quartile-based analyses are presented in online supplemental table S5.

In the restricted MACE sensitivity analysis, evidence of non-proportionality was observed for TyG, as described above. All Cox models included 991 patients. The fully adjusted model for overall cardiovascular involvement included 251 events and 17 regression parameters, and the fully adjusted model for inflammatory/lupus-related cardiovascular manifestations included 210 events and 17 regression parameters. The simplified adjusted MACE model included 41 events and six regression parameters, whereas the restricted MACE sensitivity model included 24 events and four regression parameters (online supplemental table S4).

Collectively, these findings demonstrate a consistent association between higher TyG levels and overall cardiovascular involvement and inflammatory/lupus-related cardiovascular manifestations in patients with SLE. Associations with MACE were less robust and should be interpreted cautiously given the limited number of events and the sensitivity of the estimates to outcome definition.

## Discussion

In this retrospective cohort study of patients with SLE, we found that a higher TyG index was associated with increased long-term cardiovascular involvement. This association was supported by Kaplan-Meier analyses (figure 2), restricted cubic spline analyses showing a largely linear dose–response relationship (figure 3) and multivariable Cox models (table 2 and figure 4). Importantly, after separating the heterogeneous cardiovascular composite outcome into inflammatory/lupus-related cardiovascular manifestations and MACE, the association was consistently observed for inflammatory/lupus-related manifestations, whereas the MACE findings were less robust. These findings suggest that the TyG index may serve as a marker of metabolic-immune dysregulation associated with cardiovascular involvement in SLE.

Importantly, allowing for the time-varying effects of covariates that violated the proportional hazards assumption changed the continuous TyG estimates by less than 1% for both main outcomes, supporting the robustness of the primary continuous analyses. Nevertheless, the quartile-based estimates for inflammatory/lupus-related manifestations were less precise after this adjustment and should be interpreted as secondary findings.

SLE is a complex autoimmune disease characterised by systemic inflammation, immune dysregulation and multiorgan involvement.^1 2^ CVD is a major contributor to long-term morbidity and mortality in SLE, and patients with SLE have substantially increased risks of stroke and myocardial infarction compared with the general population.^3 4^ However, traditional cardiovascular risk factors do not fully explain the excess cardiovascular burden observed in SLE.^7^ This gap highlights the need for additional biomarkers that can reflect both metabolic abnormalities and disease-related inflammatory burden.

The TyG index has emerged as a simple and practical surrogate marker of insulin resistance because it is calculated from fasting triglyceride and glucose levels, both of which are routinely available in clinical practice.^10 18^ In non-SLE populations, higher TyG levels have been associated with coronary artery calcification and atherosclerotic plaque burden,^9 12^ adverse outcomes after acute myocardial infarction^19 20^ and incident heart failure.^11^ These studies support the broader cardiovascular relevance of TyG beyond its role as a metabolic index. Our study extends this evidence to SLE, a disease in which cardiovascular involvement may arise from both cardiometabolic and immune-mediated pathways.

A major issue in studying cardiovascular outcomes in SLE is the heterogeneity of cardiovascular manifestations. Unlike conventional atherosclerotic CVD, cardiovascular involvement in SLE may include inflammatory pericardial or myocardial manifestations, valvular abnormalities, arrhythmias, pulmonary vascular disease and thrombotic or ischaemic events. Therefore, a single composite outcome may overstate or obscure distinct biological processes. To address this concern, we separated overall cardiovascular involvement into inflammatory/lupus-related cardiovascular manifestations and MACE. As shown in figure 4, TyG remained significantly associated with inflammatory/lupus-related cardiovascular manifestations after full adjustment. An association was also observed for the original MACE composite in a simplified adjusted model; however, this endpoint included heart failure, which is aetiologically heterogeneous in SLE. In a post hoc sensitivity analysis excluding heart failure, the association remained directionally positive but was attenuated after parsimonious adjustment and was no longer statistically significant. Given the small number of restricted events and evidence that the TyG effect may not have remained proportional over time, the MACE findings should be considered exploratory and should not be interpreted as evidence of an association specifically with atherosclerotic cardiovascular events (online supplemental table S3).

Our findings are also relevant to the emerging literature on TyG in immune-mediated inflammatory diseases. Previous studies have reported associations between TyG and cardiovascular risk in rheumatoid arthritis and psoriatic arthritis.^14 15^ However, evidence in SLE remains limited, and previous work has not fully addressed the time-to-event nature and phenotypic heterogeneity of cardiovascular outcomes. By applying Cox regression, Kaplan-Meier curves, and restricted cubic spline modelling, our analysis provides longitudinal evidence that higher TyG levels are associated with subsequent cardiovascular involvement in SLE. The restricted cubic spline analysis showed no evidence of significant departure from linearity (figure 3A), supporting an approximately linear association between TyG and overall cardiovascular involvement.

The baseline characteristics provide additional context for this association. Patients in the highest TyG quartile had higher frequencies of hypertension, hyperlipidaemia, diabetes, renal involvement, neuropsychiatric involvement, arthritis and pleuritis, as well as higher ESR and CRP levels and lower C3 and C4 levels (table 1). These findings suggest that elevated TyG in SLE may reflect a broader adverse metabolic–inflammatory phenotype. The attenuation of effect estimates from Model 2 to Model 3 in table 2 is also consistent with partial confounding by SLE-related manifestations and inflammatory burden. Although the association remained statistically significant after adjustment for the available SLE-related variables, these covariates cannot substitute for validated disease activity or damage indices. Accordingly, residual confounding by overall SLE activity and severity remains likely, and the observed association should not be interpreted as demonstrating risk information independent of these disease dimensions.

Several biological mechanisms may explain the observed association. Insulin resistance can promote dyslipidaemia by increasing hepatic production of very-low-density lipoprotein and impairing lipid handling, thereby contributing to a proatherogenic lipid profile.^21^ It may also impair endothelial function, reduce nitric oxide bioavailability and accelerate vascular injury.^22^ In addition, insulin resistance has been linked to chronic inflammatory signalling, including activation of nuclear factor kappa-light-chain-enhancer of activated B cell pathways and increased production of inflammatory cytokines.^23 24^ In SLE, these metabolic disturbances may interact with chronic immune activation and complement consumption, potentially amplifying vascular inflammation and injury. Prior work has also suggested that insulin resistance and oxidative stress may be connected to SLE-related coronary disease.^25 26^ Therefore, TyG may serve as an accessible marker reflecting the convergence of metabolic dysfunction, inflammation and immune-mediated cardiovascular vulnerability.

This study has several strengths. First, it included a relatively large SLE cohort with long-term follow-up, allowing time-to-event analyses rather than cross-sectional or logistic models. Second, the TyG index was recalculated using standard unit conversions, and its association with outcomes was examined both continuously and by quartiles. Third, we performed outcome-specific analyses to distinguish inflammatory/lupus-related cardiovascular manifestations from MACE and conducted an additional sensitivity analysis excluding heart failure from the MACE composite, directly addressing the heterogeneity of cardiovascular involvement in SLE. Fourth, we adjusted for both traditional cardiometabolic risk factors and several SLE-related variables, including organ involvement, inflammatory markers, complement levels and glucocorticoid use. Fifth, we explicitly evaluated the proportional hazards assumption and performed extended Cox sensitivity analyses using stratification and time-dependent coefficients where appropriate.

Several limitations should be acknowledged. First, the retrospective single-centre design limits causal inference and generalisability. Second, residual confounding by SLE disease activity and cumulative disease severity is an important limitation. Although the multivariable models incorporated available organ manifestations, ESR, CRP, complement levels and glucocorticoid use as clinical and serological proxies, standardised disease activity and damage measures, including the SLE disease activity index, Physician Global Assessment and the Systemic Lupus International Collaborating Clinics/ACR Damage Index, were not uniformly available. These proxy variables cannot fully capture the multidimensional and longitudinal nature of SLE activity or accumulated organ damage. This limitation is particularly relevant because higher TyG quartiles were associated with several markers of greater inflammatory and organ involvement at baseline. Therefore, the observed TyG–cardiovascular association may partly reflect underlying SLE activity or severity, and our findings should not be interpreted as establishing TyG as a predictor independent of these disease dimensions. Data on disease duration, cumulative glucocorticoid exposure, antiphospholipid antibodies, body mass index, statin use, diet and physical activity were also incomplete or unavailable. In addition, no formal correction for multiple testing was applied. Because several secondary and exploratory analyses were performed, these findings should be interpreted cautiously and require external validation. Third, TyG was assessed at baseline only, and longitudinal changes during follow-up were unavailable. Repeated measurements may better capture cumulative metabolic burden and dynamic metabolic changes over time and should be evaluated in future studies. Fourth, although time-to-event analyses were performed, some cardiovascular abnormalities may have been subclinical or present before baseline TyG measurement; therefore, temporality cannot be fully established in all cases. Fifth, the original MACE composite included heart failure, the aetiology of which is heterogeneous in SLE and could not be uniformly adjudicated as ischaemic or atherosclerotic in this retrospective cohort. Although a post hoc sensitivity analysis excluding heart failure was performed, only 24 restricted MACE events remained, resulting in imprecise estimates and limited statistical power. The limited event-to-parameter ratio in the MACE and restricted MACE models further supports interpreting these analyses as exploratory. In addition, evidence of non-proportionality for TyG was observed in this restricted analysis. Accordingly, the MACE findings should be interpreted as exploratory and require confirmation using prospectively adjudicated cardiovascular endpoints. Quartile-based estimates for inflammatory/lupus-related cardiovascular manifestations were less precise in the proportional hazards-adjusted sensitivity analysis, and these secondary categorical findings should therefore be interpreted cautiously.

In conclusion, higher TyG index levels were associated with increased long-term cardiovascular involvement in patients with SLE. The association was also observed for inflammatory/lupus-related cardiovascular manifestations and remained evident when TyG was analysed as a continuous exposure after accounting for non-proportional covariate effects. Findings for MACE were less robust and should be interpreted cautiously because of the limited number of events and the sensitivity of the estimates to outcome definition. These findings suggest that TyG may serve as a readily available marker associated with cardiovascular involvement in SLE. Further prospective multicentre studies incorporating standardised disease activity indices, adjudicated cardiovascular outcomes and repeated TyG measurements are needed to validate these findings and clarify the underlying mechanisms.

## Supplementary material

10.1136/lupus-2026-002141 online supplemental file 1

## Data Availability

All data relevant to the study are included in the article or uploaded as supplementary information.
