# Supplementary material for: Long-term association of the triglyceride-glucose index with cardiovascular involvement in systemic lupus erythematosus: a retrospective cohort study
Source: Lupus Sci Med. 2026 Sep 12;13(2):e002141. doi: 10.1136/lupus-2026-002141 (PMC13583751; doi:10.1136/lupus-2026-002141)
Supplement: Supplementary file 1 [file lupus-13-2-s001.docx]

# 1Supplementary Table S1A. Cox regression analyses for inflammatory/lupus-related cardiovascular manifestations according to the TyG index

| **Exposure** | **Model 1 HR (95% CI)** | **Model 1 P value** | **Model 2 HR (95% CI)** | **Model 2 P value** | **Model 3 HR (95% CI)** | **Model 3 P value** |
| --- | --- | --- | --- | --- | --- | --- |
| Continuous TyG | 1.62 (1.32–1.99) | <0.001 | 1.62 (1.30–2.02) | <0.001 | 1.36 (1.07–1.73) | 0.012 |
| TyG quartiles |  |  |  |  |  |  |
| Q1 | Ref. |  | Ref. |  | Ref. |  |
| Q2 | 1.44 (0.93–2.23) | 0.100 | 1.47 (0.95–2.28) | 0.087 | 1.20 (0.77–1.89) | 0.421 |
| Q3 | 1.86 (1.22–2.83) | 0.004 | 1.86 (1.22–2.85) | 0.004 | 1.56 (1.01–2.40) | 0.045 |
| Q4 | 2.32 (1.53–3.51) | <0.001 | 2.29 (1.49–3.52) | <0.001 | 1.55 (0.99–2.42) | 0.053 |
| P for trend |  | <0.001 |  | <0.001 |  | 0.030 |

Notes: Model 1 adjusted for age and sex. Model 2 additionally adjusted for smoking, hypertension, hyperlipidaemia, and diabetes mellitus. Model 3 additionally adjusted for renal involvement, neuropsychiatric involvement, haematologic involvement, arthritis, pleuritis, ESR, CRP, C3, C4, and glucocorticoid use. Abbreviations: TyG, triglyceride–glucose index; HR, hazard ratio; CI, confidence interval; ESR, erythrocyte sedimentation rate; CRP, C-reactive protein.

# Supplementary Table S1B. Cox regression analyses for major adverse cardiovascular events according to the TyG index

| **Exposure** | **Model 1 HR (95% CI)** | **Model 1 P value** | **Simplified adjusted HR (95% CI)** | **Simplified adjusted P value** |
| --- | --- | --- | --- | --- |
| Continuous TyG | 2.27 (1.46–3.54) | <0.001 | 1.82 (1.13–2.92) | 0.014 |
| TyG quartiles |  |  |  |  |
| Q1 | Ref. |  | Ref. |  |
| Q2 | 2.15 (0.66–6.98) | 0.205 | 2.29 (0.70–7.53) | 0.172 |
| Q3 | 2.42 (0.74–7.97) | 0.146 | 2.04 (0.61–6.85) | 0.247 |
| Q4 | 5.63 (1.89–16.75) | 0.002 | 4.67 (1.51–14.43) | 0.007 |
| P for trend |  | <0.001 |  | 0.006 |

Notes: Model 1 adjusted for age and sex. Because only 41 MACE events occurred, the simplified adjusted model included age, sex, hypertension, hyperlipidaemia, and diabetes mellitus. Abbreviations: TyG, triglyceride–glucose index; HR, hazard ratio; CI, confidence interval; MACE, major adverse cardiovascular events.

# Supplementary Table S2. Tests of the proportional hazards assumption based on scaled Schoenfeld residuals

Table S2A. Overall cardiovascular involvement (Model 3)

| Variable | PH test P value |
| --- | --- |
| TyG | 0.552 |
| Age | 0.008 |
| Sex | 0.450 |
| Smoking | 0.719 |
| Hypertension | 0.115 |
| Hyperlipidaemia | 0.001 |
| Diabetes | 0.746 |
| Kidney involvement | 0.436 |
| Neuropsychiatric involvement | 0.617 |
| Haematologic involvement | 0.174 |
| Arthritis | 0.124 |
| Pleuritis | 0.016 |
| ESR | 0.370 |
| CRP | 0.636 |
| C3 | 0.520 |
| C4 | 0.394 |
| Glucocorticoid use | 0.244 |
| Global test | 0.010 |

Table S2B. Inflammatory/lupus-related cardiovascular manifestations (Model 3)

| Variable | PH test P value |
| --- | --- |
| TyG | 0.821 |
| Age | 0.006 |
| Sex | 0.678 |
| Smoking | 0.461 |
| Hypertension | 0.024 |
| Hyperlipidaemia | 0.006 |
| Diabetes | 0.733 |
| Kidney involvement | 0.723 |
| Neuropsychiatric involvement | 0.595 |
| Haematologic involvement | 0.070 |
| Arthritis | 0.147 |
| Pleuritis | 0.004 |
| ESR | 0.135 |
| CRP | 0.771 |
| C3 | 0.027 |
| C4 | 0.845 |
| Glucocorticoid use | 0.322 |
| Global test | 0.001 |

Table S2C. Major adverse cardiovascular events (MACE) (simplified adjusted model)

| Variable | PH test P value |
| --- | --- |
| TyG | 0.180 |
| Age | 0.982 |
| Sex | 0.105 |
| Hypertension | 0.681 |
| Hyperlipidaemia | 0.008 |
| Diabetes | 0.828 |
| Global test | 0.147 |

A two-sided P value <0.05 indicated evidence against the proportional hazards assumption. The global test jointly evaluated the proportional hazards assumption for the entire model.

Abbreviations: PH, proportional hazards; ESR, erythrocyte sedimentation rate; CRP, C-reactive protein; MACE, major adverse cardiovascular events. The proportional hazards assumption was evaluated using Schoenfeld residuals.

Supplementary Table S3. Sensitivity analysis of cardiovascular events excluding heart failure.

| **Outcome** | **Model** | **Events** | **HR per 1-unit increase in TyG (95% CI)** | **P value** |
| --- | --- | --- | --- | --- |
| MACE excluding HF | Crude | 24 | 2.07 (1.15–3.72) | 0.015 |
| MACE excluding HF | Model 1 | 24 | 2.08 (1.14–3.81) | 0.018 |
| MACE excluding HF | Parsimonious adjusted model | 24 | 1.57 (0.85–2.89) | 0.147 |

Notes: MACE excluding heart failure comprised cardiovascular death, cerebral infarction, and myocardial infarction. Model 1 adjusted for age and sex. The parsimonious adjusted model additionally included hypertension. Because only 24 events occurred and the proportional hazards assumption showed evidence of deviation for TyG in the parsimonious adjusted model (Schoenfeld residual test P=0.029), this sensitivity analysis was considered exploratory.

Supplementary Table S4. Sample size, event count, and model complexity in Cox regression analyses

| **Outcome** | **Model** | **N in model** | **Events in model** | **Regression parameters** | **Events per parameter** | **Model covariates** |
| --- | --- | --- | --- | --- | --- | --- |
| Overall cardiovascular involvement | Model 1 | 991 | 251 | 3 | 83.67 | TyG + age + sex |
| Overall cardiovascular involvement | Model 2 | 991 | 251 | 7 | 35.86 | TyG + age + sex + smoking + hypertension + hyperlipidaemia + diabetes mellitus |
| Overall cardiovascular involvement | Model 3 | 991 | 251 | 17 | 14.76 | TyG + age + sex + smoking + hypertension + hyperlipidaemia + diabetes mellitus + renal involvement + neuropsychiatric involvement + haematologic involvement + arthritis + pleuritis + ESR + CRP + C3 + C4 + glucocorticoid use |
| Inflammatory/lupus-related cardiovascular manifestations | Fully adjusted model | 991 | 210 | 17 | 12.35 | TyG + age + sex + smoking + hypertension + hyperlipidaemia + diabetes mellitus + renal involvement + neuropsychiatric involvement + haematologic involvement + arthritis + pleuritis + ESR + CRP + C3 + C4 + glucocorticoid use |
| Major adverse cardiovascular events | Simplified adjusted model | 991 | 41 | 6 | 6.83 | TyG + age + sex + hypertension + hyperlipidaemia + diabetes mellitus |
| MACE excluding heart failure | Parsimonious adjusted sensitivity model | 991 | 24 | 4 | 6.00 | TyG + age + sex + hypertension |

Notes: The number of regression parameters refers to the number of estimated coefficients in each Cox model, including the TyG exposure term. Events per parameter were calculated as the number of events divided by the number of regression parameters. MACE, major adverse cardiovascular events; TyG, triglyceride–glucose index.

Supplementary Table S5. Sensitivity analyses of the associations between the TyG index and cardiovascular outcomes after accounting for non-proportional covariate effects

Table S5A. Overall cardiovascular involvement

| **Exposure** | **Original fully adjusted model HR (95% CI)** | **P value** | **PH-adjusted sensitivity model HR (95% CI)** | **P value** |
| --- | --- | --- | --- | --- |
| Continuous TyG | 1.357 (1.092–1.687) | 0.006 | 1.366 (1.095–1.705) | 0.006 |
| TyG quartiles |  |  |  |  |
| Q1 | Ref. |  | Ref. |  |
| Q2 | 1.285 (0.841–1.963) | 0.246 | 1.201 (0.785–1.839) | 0.398 |
| Q3 | 1.548 (1.029–2.330) | 0.036 | 1.473 (0.978–2.220) | 0.064 |
| Q4 | 1.682 (1.115–2.538) | 0.013 | 1.616 (1.069–2.443) | 0.023 |
| P for trend |  | 0.009 |  | 0.014 |

Table S5B. Inflammatory/lupus-related cardiovascular manifestations

| **Exposure** | **Original fully adjusted model HR (95% CI)** | **P value** | **PH-adjusted sensitivity model HR (95% CI)** | **P value** |
| --- | --- | --- | --- | --- |
| Continuous TyG | 1.363 (1.072–1.733) | 0.012 | 1.354 (1.055–1.737) | 0.017 |
| TyG quartiles |  |  |  |  |
| Q1 | Ref. |  | Ref. |  |
| Q2 | 1.204 (0.766–1.890) | 0.421 | 1.190 (0.755–1.876) | 0.453 |
| Q3 | 1.559 (1.010–2.404) | 0.045 | 1.504 (0.971–2.328) | 0.067 |
| Q4 | 1.550 (0.994–2.416) | 0.053 | 1.459 (0.925–2.304) | 0.105 |
| P for trend |  | 0.030 |  | 0.065 |

Notes: The original fully adjusted models included age, sex, smoking, hypertension, hyperlipidaemia, diabetes mellitus, renal involvement, neuropsychiatric involvement, haematologic involvement, arthritis, pleuritis, ESR, CRP, C3, C4, and glucocorticoid use.

In the proportional hazards-adjusted model for overall cardiovascular involvement, age was modelled using a time-dependent coefficient through an interaction with centred log follow-up time, and the model was stratified by hyperlipidaemia and pleuritis. In the corresponding model for inflammatory/lupus-related cardiovascular manifestations, age and C3 were modelled using time-dependent coefficients, and the model was stratified by hypertension, hyperlipidaemia, and pleuritis.

The relative changes in the HR for continuous TyG between the original and proportional hazards-adjusted models were +0.65% for overall cardiovascular involvement and −0.67% for inflammatory/lupus-related cardiovascular manifestations.

Abbreviations: TyG, triglyceride–glucose index; PH, proportional hazards; HR, hazard ratio; CI, confidence interval; ESR, erythrocyte sedimentation rate; CRP, C-reactive protein.

Supplementary Figure 1. Proportional hazards diagnostics for overall cardiovascular involvement

Scaled Schoenfeld residual plots are presented for (A) the TyG index, (B) age, (C) hyperlipidaemia, and (D) pleuritis in the fully adjusted Cox model for overall cardiovascular involvement. Open circles represent scaled Schoenfeld residuals. The solid curve represents the smoothed estimate of the time-varying coefficient, β(t), and the dashed curves represent the approximate 95% confidence bands. The horizontal dotted line represents the constant coefficient estimated under the conventional proportional hazards model. The x-axis represents follow-up time in days. P values were obtained from variable-specific Schoenfeld residual tests. There was no evidence that the proportional hazards assumption was violated for TyG (P=0.552), whereas age, hyperlipidaemia, and pleuritis showed evidence of non-proportional effects. The global test for the fully adjusted model was significant (P=0.010).

Abbreviations: TyG, triglyceride–glucose index.

Supplementary Figure 2. Proportional hazards diagnostics for inflammatory/lupus-related cardiovascular manifestations.

Scaled Schoenfeld residual plots are presented for (A) the TyG index, (B) age, (C) hypertension, (D) hyperlipidaemia, (E) pleuritis, and (F) complement C3 in the fully adjusted Cox model for inflammatory/lupus-related cardiovascular manifestations. Open circles represent scaled Schoenfeld residuals. The solid curve represents the smoothed estimate of the time-varying coefficient, β(t), and the dashed curves represent the approximate 95% confidence bands. The horizontal dotted line represents the constant coefficient estimated under the conventional proportional hazards model. The x-axis represents follow-up time in days. P values were obtained from variable-specific Schoenfeld residual tests. There was no evidence that the proportional hazards assumption was violated for TyG (P=0.821), whereas age, hypertension, hyperlipidaemia, pleuritis, and C3 showed evidence of non-proportional effects. The global test for the fully adjusted model was significant (P=0.001).

Abbreviations: TyG, triglyceride–glucose index; C3, complement component 3.
